# TRPC expression in human periodontal ligament cells and the periodontal tissue of periodontitis mice: a preliminary study

**DOI:** 10.1186/s42826-023-00171-6

**Published:** 2023-09-01

**Authors:** Aeryun Kim, Ae Ri Kim, Yeong-Eui Jeon, Yun‑Jung Yoo, Yu-Mi Yang, Eun‑Jung Bak

**Affiliations:** 1 Department of Oral Biology, Yonsei University College of Dentistry, 134 Sinchon Dong, Seodaemun-gu, Seoul, 03722 Republic of Korea; 2 BK21 FOUR Project, Yonsei University College of Dentistry, Seoul, 03722 Republic of Korea; 3 Present Address: Oral Health Research Institute, Apple Tree Dental Hospital, Bucheon, 14642 Republic of Korea

**Keywords:** PDL cells, Periodontitis, TRPC3, TRPC6, Osteoblasts

## Abstract

**Background:**

Transient receptor potential canonical (TRPC) channels are non-selective cationic channels with permeability to Ca^2+^ and Na^+^. Despite their importance, there are currently few studies on TRPC in the periodontal ligament (PDL) and bone cells in the dental field. To provide biological information regarding TRPC in PDL cells and periodontal tissue, we evaluated TRPC channels expression in the osteoblast differentiation of PDL cells and periodontitis-induced tissue. Human PDL cells were cultured in osteogenic differentiation media for 28 days, and the expression of Runx2, osteocalcin (OCN), and TRPC1, 3, 4, and 6 was evaluated by real-time PCR. In ligature-induced periodontitis mice, the alveolar bone and osteoid areas, the osteoclast number, and the expression of Runx2, OCN, TRPC3, and TRPC6 was evaluated by H&E staining, TRAP staining, and immunohistochemistry, respectively.

**Results:**

In the PDL cell differentiation group, TRPC6 expression peaked on day 7 and TRPC3 expression generally increased during differentiation. During the 28 days of periodontitis progression, alveolar bone loss and osteoclast numbers increased compared to the control group during the experimental period and the osteoid area increased from day 14. TRPC6 expression in the periodontitis group increased in the PDL area and in the osteoblasts compared to the control group, whereas TRPC3 expression increased only in the PDL area on days 7 and 28.

**Conclusions:**

These results indicate changes of TRPC3 and TRPC6 expression in PDL cells that were differentiating into osteoblasts and in periodontitis-induced tissue, suggesting the need for research on the role of TRPC in osteoblast differentiation or periodontitis progression.

## Background

Mammalian transient receptor potential (TRP) channels are composed of seven families, including of TRPC (canonical), TRPM (melastatin), TRPV (vanilloid), TRPML (mucolipin), TRPP (polycystin), TRPA (ankyrin), and TRPN (no mechanoreceptor potential C) [1]. In human, TRPC, TRPM, and TRPV consist of TRPC1–7, TRPM1–8, and TRPV1–6 channels, respectively [2]. The TRPC subfamily can be grouped based on sequence homology as TRPC1, TRPC4/C5, TRPC3/C6/C7, and TRPC2 (a pseudogene in humans) [3]. They are the most common non-selective cation channels with permeability to Ca^2+^, Na^+^, and K^+^ and are activated by various stimuli such as intracellular and extracellular signals, and mechanical and osmotic stress [4, 5]. Although the association of TRPML, TRPP, TRPA, and TRPN with human diseases has not yet been fully elucidated, TRPC, TRPM, and TRPV are known to be involved [5]. TRPC4 mutation is associated with the prevention of myocardial infarction in diabetes [6], and TRPC6 mutation is associated with familial focal segmental glomerulosclerosis [7] and idiopathic pulmonary hypertension [8]. Furthermore, the in vivo genetic deletion or pharmacologic inhibition of TRPC3 and/or TRPC6 diminished renal fibrosis in a unilateral ureteral obstruction model [9, 10]. These findings suggest that biological information about TRPC may benefit on the understanding of cellular responses to several stimuli as well as therapeutic approaches to human diseases.

Periodontal tissue is composed of the periodontal ligament (PDL), alveolar bone, gingiva, and cementum [11]. Among them, the PDL maintains the homeostasis of adjacent tissues such as alveolar bone and teeth against the extracellular environment and stimuli throughout life [12, 13] and is composed of heterogeneous cells such as fibroblasts, osteoblasts, and cementoblasts. PDL fibroblasts have functions in sensing and mediating mechanical load and triggering pro-inflammatory responses [14, 15]. Studies have been conducted on the TRP-mediated regulation of PDL cells and osteoblasts. A previous report showed that Ca^2+^ entry via TRPV4 and TRPM3 activated through hypotonic stress increased RANKL expression in PDL cells [16]. TRPC, TRPM, and TRPV channels are expressed in human and murine osteoblastic cells, and TRPC and TRPM channels are involved in osteoblast proliferation [5]. TRPM7 regulates osteoblast proliferation and intracellular ion homeostasis through the inflow of Ca^2+^ and Mg^2+^ [17]. TRPC3 mediates the regulation of 1α,25(OH)_2_D_3_-induced capacitative calcium entry (CCE) in osteoblast-like cells through the formation of a complex with the vitamin D receptor [18]. The information provided by previous studies has emphasized the role of TRP-mediated regulation in PDL cells and osteoblasts. However, there is still a lack of research on both the TRPC-mediated regulatory mechanism and the expression in PDL cells and osteoblasts.

TRP channels in the oral cavity have been reported to be related to pain transduction in pulpitis, alveolar bone destruction by excessive mechanical force, dysfunction of salivary glands, and periodontal inflammatory responses [19–22]. Periodontitis, a typical periodontal disease, is a chronic disease that can lead to tooth loss by alveolar bone resorption due to continued inflammation. During periodontitis progression, an increase in pro-inflammatory cytokines, rearrangement of PDL, and changes in the number of bone cells (osteoblasts, osteoclasts, and osteocytes) occur [23, 24]. PDL cells play an important role in the pathogenesis of periodontitis by producing pro-inflammatory cytokines [25]. PDL cells differentiate into osteoblasts and cementoblasts, and osteoblasts secrete bone matrix to eventually differentiate into osteocytes [26]. Therefore, PDL cells and osteoblasts are considered to play a pivotal role in the regeneration of alveolar bone. Investigation of TRPC expression in PDL cells and osteoblasts in periodontitis-induced tissue provides evidence that TRPC may be involved in periodontitis. The ligature-induced periodontitis model is a valuable tool that demonstrates the occurrence of periodontal inflammation and alveolar bone resorption due to the accumulation of periodontopathogens [27]. In this study, we evaluated TRPCs expression in PDL cells during a 28-day period of osteoblast differentiation. The expression of TRPC3 and TRPC6 was investigated in mice periodontal tissue during a 28-day periodontitis induction period.

## Results

### Expression of osteoblast differentiation factors in human PDL cells during osteoblast differentiation

**Fig. 1:**
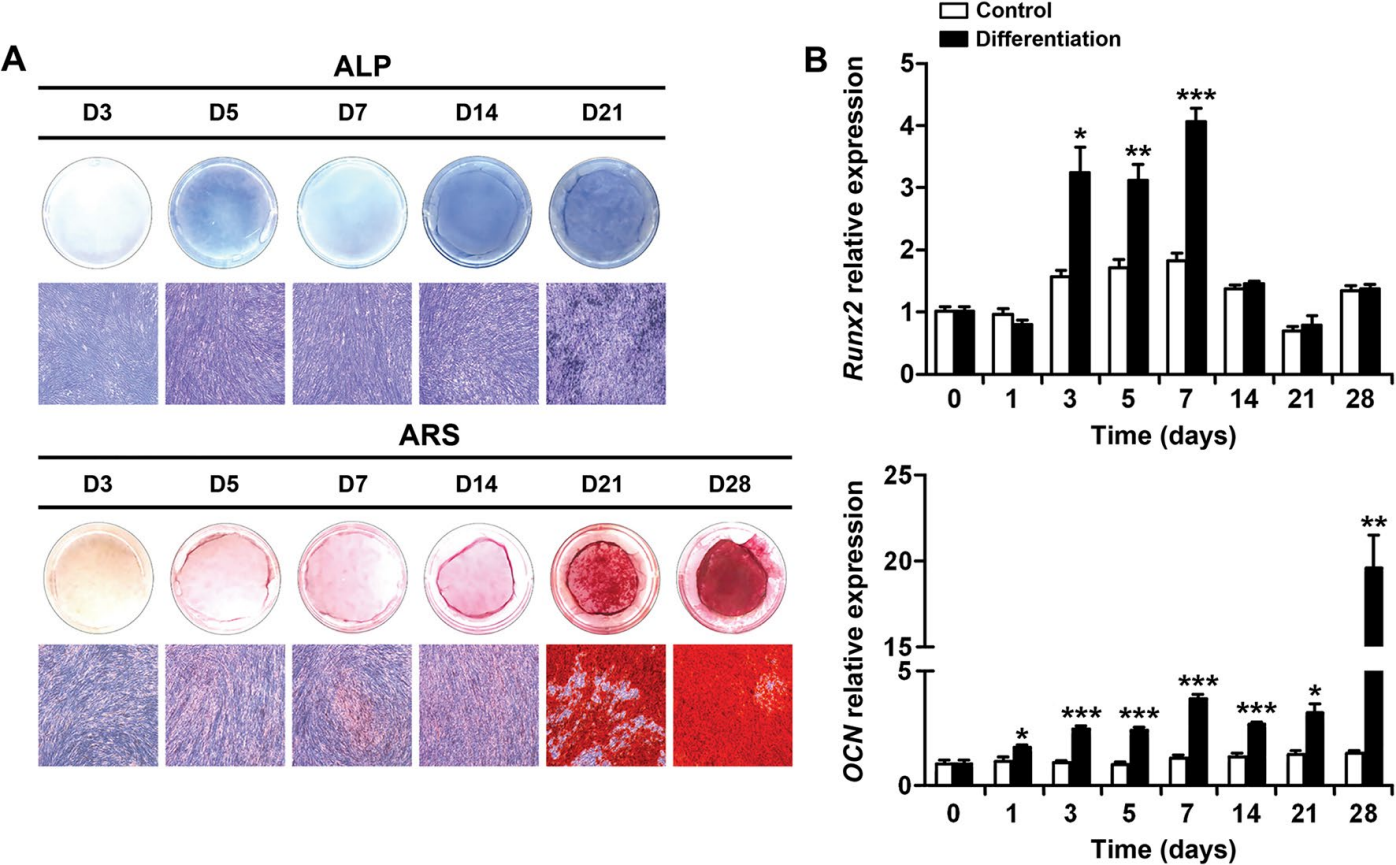
Expression of osteoblast differentiation factors during osteoblast differentiation of human PDL cells. **A** Representative images of ALP staining and ARS staining during the differentiation period. **B** mRNA expression of osteoblast differentiation factors, Runx2 and OCN during the differentiation period. Data are presented as the mean ± SE. *p < 0.05 versus control, **p < 0.01 versus control, ***p < 0.001 versus control. ALP, alkaline phosphatase; ARS, alizarin red S; D, day; OCN, osteocalcin

Alkaline phosphatase (ALP) and alizarin red S (ARS) staining were performed to determine the degree of osteoblast differentiation during a 28-day differentiation period of PDL cells (Fig. 1A). ALP staining in the differentiation group became stronger from day 5, and ARS staining became stronger from day 21. Next, the mRNA expression levels of osteoblast differentiation factors Runx2 and osteocalcin (OCN) were evaluated by real-time PCR (Fig. 1B). Runx2 expression was higher in the differentiation group than in the control group from days 3 to 7. OCN expression was higher in the differentiated group than in the control group and peaked on day 28.

### Expression of TRPC channels in human PDL cells during osteoblast differentiation

**Fig. 2:**
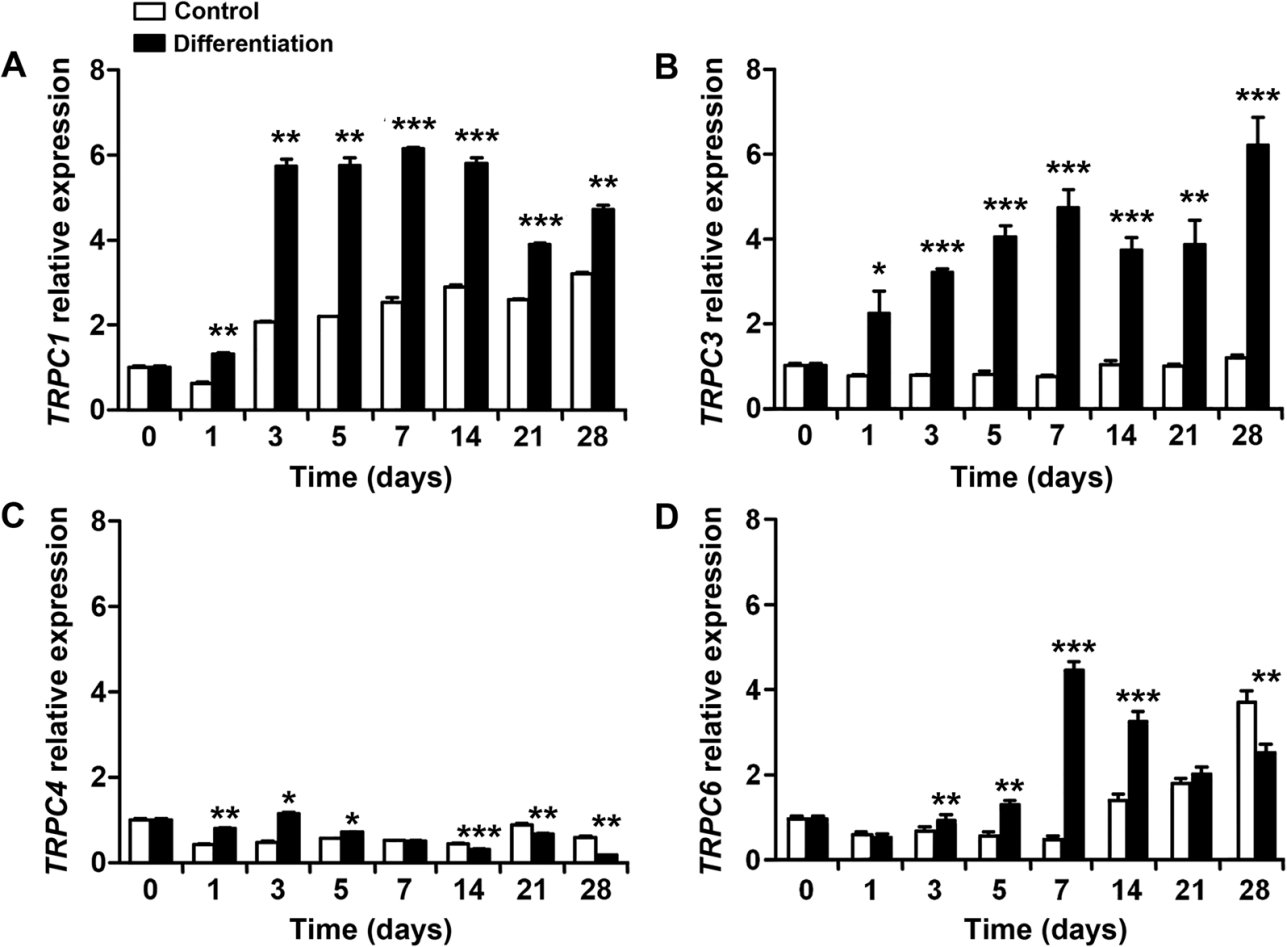
mRNA expression of TRPC channels during the osteoblast differentiation of human PDL cells. **A** TRPC1. **B** TRPC3. **C** TRPC4. **D** TRPC6. Data are presented as the mean ± SE. *p < 0.05 versus control, **p < 0.01 versus control, ***p < 0.001 versus control. TRPC, transient receptor potential canonical

Real-time PCR was performed to evaluate the mRNA expression levels of TRPC1, 3, 4, and 6 in PDL cells during osteoblast differentiation (Fig. 2). TRPC3 expression showed a continuous increase during the differentiation period compared to the control group, and TRPC6 expression peaked on day 7. The expression of TRPC1 was most evident from days 3 to 14 of the differentiation period, and the expression of TRPC4 was weaker than that of other TRPC channels.

### Alveolar bone loss during periodontitis progression

**Fig. 3:**
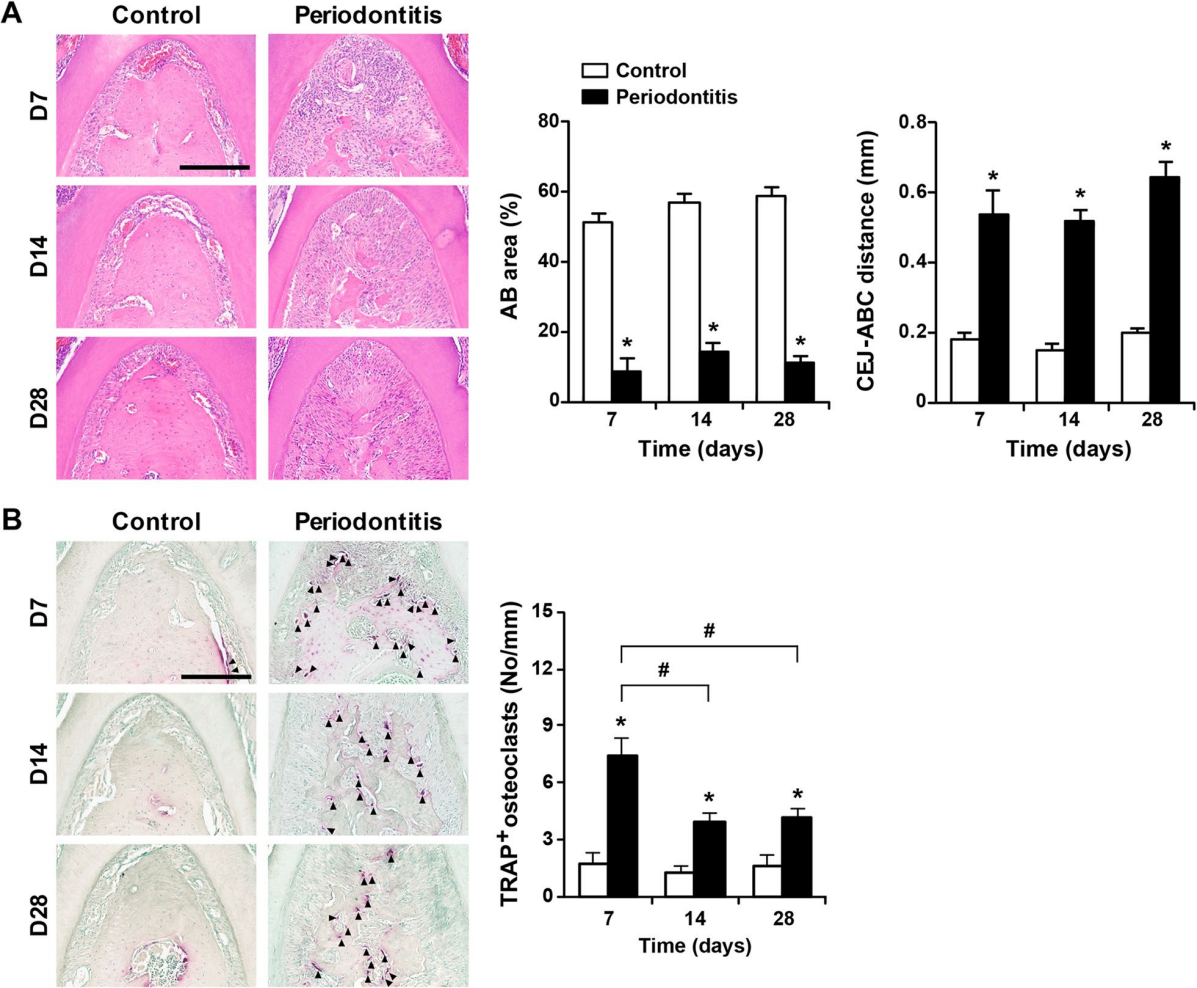
Alveolar bone loss during periodontitis progression. **A** Representative images of furcation and alveolar bone loss in mice with periodontitis. Alveolar bone loss was determined by the alveolar bone area in the furcation area (control group; D7 n = 7, D14 n = 7, D28 n = 10, and periodontitis group; D7 n = 7, D14 n = 9, D28 n = 11) and the CEJ-ABC distance in the distal area (control group; D7 n = 7, D14 n = 7, D28 n = 10, and periodontitis group; D7 n = 7, D14 n = 9, D28 n = 10). Scale bar = 200 μm. **B** Representative images and osteoclast formation in mice with periodontitis. Osteoclast formation was determined by counting TRAP-positive osteoclasts (control group; D7 n = 8, D14 n = 8, D28 n = 8, and periodontitis group; D7 n = 7, D14 n = 9, D28 n = 12). Black arrowheads indicate representative TRAP-positive osteoclasts. Scale bar = 200 μm. Data are presented as the mean ± SE. *p < 0.05 versus control. #*p* < 0.017 versus days. AB, alveolar bone; ABC, alveolar bone crest; CEJ, cementoenamel junction; No, number; TRAP, tartrate-resistant acid phosphatase

The alveolar bone level and number of osteoclasts in the mandibular first molars were measured by hematoxylin and eosin (H&E) staining and tartrate-resistant acid phosphatase (TRAP) staining, respectively, to determine alveolar bone loss during periodontitis progression (Fig. 3). The alveolar bone area decreased over 28 days, and the cementoenamel junction (CEJ)-alveolar bone crest (ABC) distance increased in the periodontitis group compared to the control group (Fig. 3A). The number of osteoclasts increased during the 28 days compared to the control group, peaking on day 7 (Fig. 3B). The number of osteoclasts decreased on days 14 and 28 compared to day 7.

### Alveolar bone formation during periodontitis progression

**Fig. 4:**
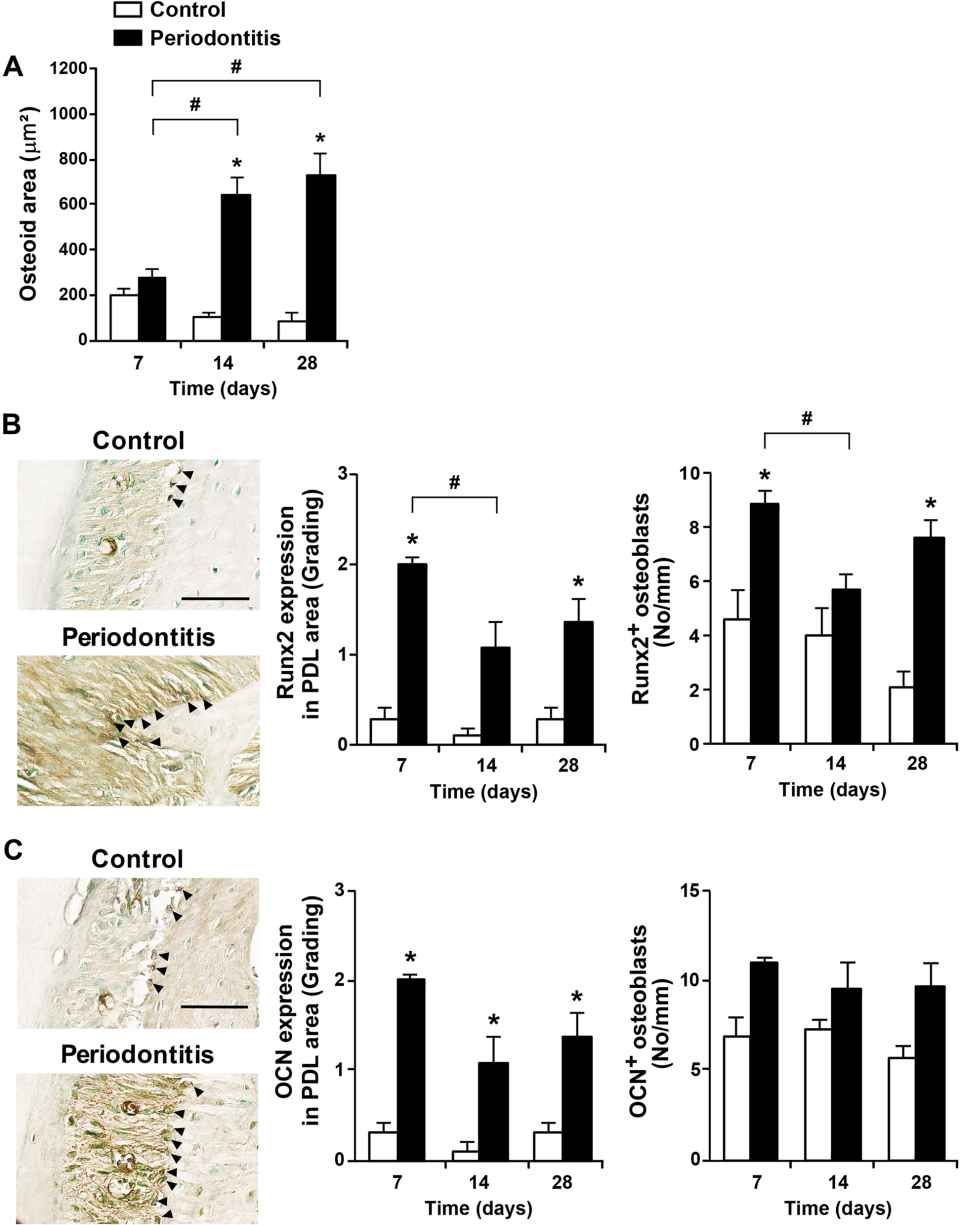
Alveolar bone formation during periodontitis progression. **A** Osteoid formation in mice with periodontitis. (control group; D7 n = 8, D14 n = 8, D28 n = 10, and periodontitis group; D7 n = 8, D14 n = 9, D28 n = 11). **B** Representative images on day 28 and the Runx2 expression (control group; D7 n = 8, D14 n = 6, D28 n = 8, and periodontitis group; D7 n = 7, D14 n = 10, D28 n = 8). Black arrowheads indicate representative Runx2-positive osteoblasts. Scale bar = 50 μm.** C** Representative images on day 28 and the OCN expression (control group; D7 n = 5, D14 n = 5, D28 n = 5, and periodontitis group; D7 n = 7, D14 n = 7, D28 n = 7). Black arrowheads indicate representative OCN-positive osteoblasts. Scale bar = 50 μm. Data are presented as the mean ± SE. *p < 0.05 versus control. #*p* < 0.017 versus days. No, number; OCN, osteocalcin; PDL, periodontal ligament

Next, osteoid areas and Runx2 and OCN expression were measured to evaluate alveolar bone formation and expression of osteoblast differentiation factors in periodontal tissue (Fig. 4). Osteoid areas increased in the periodontitis group compared to the control group on days 14 and 28. The periodontitis group also showed an increase in osteoid areas on days 14 and 28 compared to day 7 (Fig. 4A). The Runx2 and OCN expression was determined in the PDL area and osteoblasts of the periodontal tissue by immunohistochemical staining (Fig. 4B and C). Runx2 expression of both the PDL area and the osteoblasts in the periodontitis group was higher on days 7 and 28 than in the control group, with a marked increase on day 7 (Fig. 4B). During the experimental period, OCN expression in the PDL area was higher in the periodontitis group than in the control group, and there was no difference in expression in osteoblasts between the control group and the periodontitis group (Fig. 4C).

### Expression of TRPC3 and TRPC6 during periodontitis progression

**Fig. 5:**
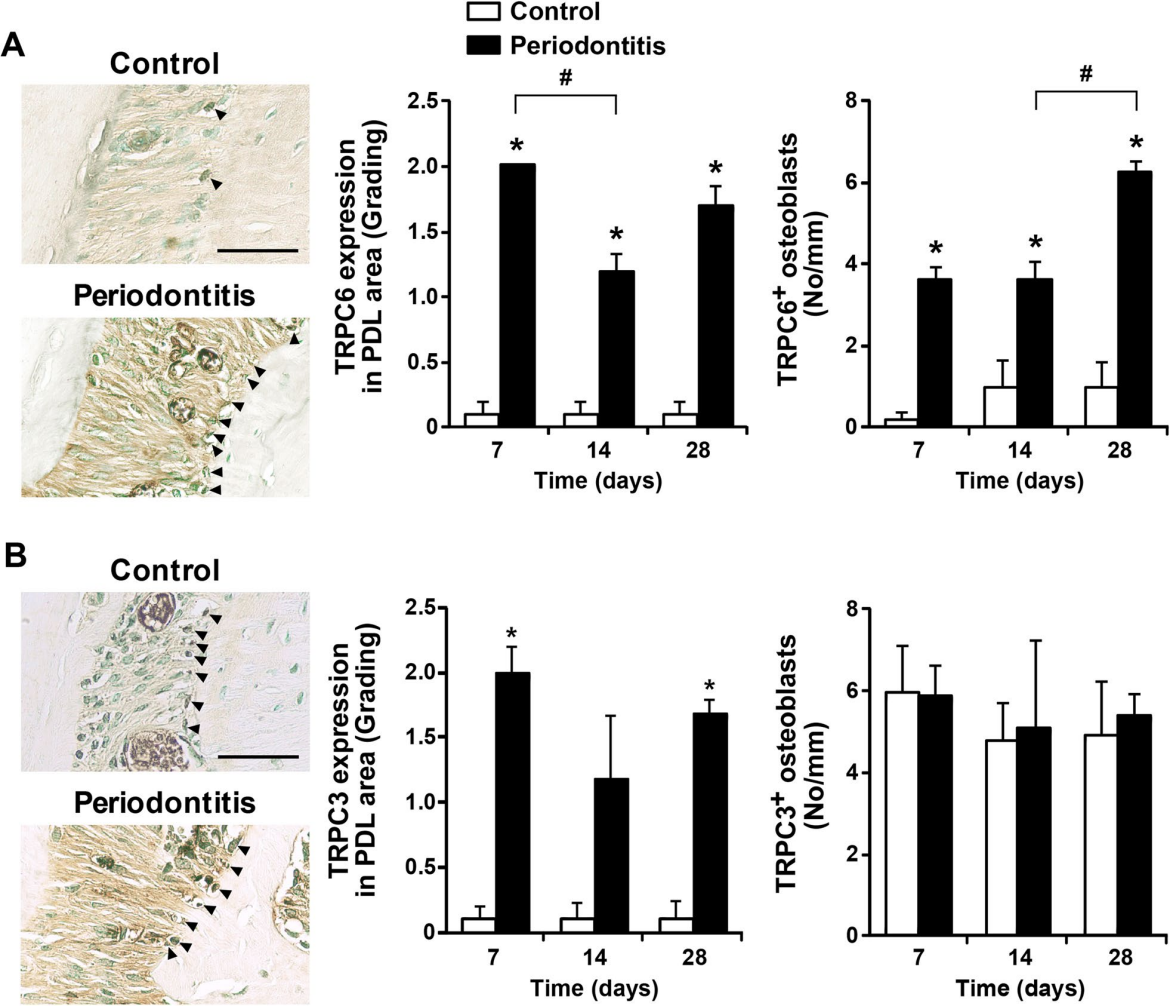
TRPC6 and TRPC3 expression during periodontitis progression. **A** Representative images on day 28 and TRPC6 expression (control group; D7 n = 5, D14 n = 5, D28 n = 4, and periodontitis group; D7 n = 3, D14 n = 3, D28 n = 5). Black arrowheads indicate representative TRPC3-positive osteoblasts. **B** Representative images on day 28 and TRPC3  expression (control group; D7 n = 5, D14 n = 5, D28 n = 5, and periodontitis group; D7 n = 5, D14 n = 8, D28 n = 8). Black arrowheads indicate representative TRPC6-positive osteoblasts. Scale bar = 50 μm. Data are presented as the mean ± SE. *p < 0.05 versus control. No, number; PDL, periodontal ligament; TRPC, transient receptor potential canonical

TRPC3 has been considered to be a molecular component of 1α,25(OH)_2_D_3_-activated CCE in osteoblast-like cells [18]. TRPC6 has also been implicated in numerous biological processes, including the regulation of endothelial permeability and leukocyte transmigration [28]. Based on the results of previous studies and the expression of TRPC channels during osteoblast differentiation of PDL cells in this study, we focused on the expression of TRPC3 and TRPC6 during periodontitis progression, which was evaluated by immunohistochemical staining (Fig. 5). TRPC3 expression in the PDL area was higher in the periodontitis group on days 7 and 28 than in the control group, and there was no difference in expression in osteoblasts between the control group and the periodontitis group (Fig. 5A). The TRPC6 expression of both the PDL area and the osteoblasts in the periodontitis group was consistently higher than that of the control group (Fig. 5B). TRPC6 expression in the PDL area of the periodontitis group on day 14 was lower than that on day 7, and TRPC6 expression in osteoblasts of the periodontitis group on day 28 was higher than that on day 14.

## Discussion

We evaluated the expression of osteoblast differentiation factors and TRPCs in commercially available human PDL cells cultured in osteogenic differentiation conditions. The results showed that during the differentiation of human PDL cells, the expression of Runx2, a regulator of osteoblast differentiation, significantly increased on days 3–7, and the expression of OCN, a late-stage marker of osteoblast differentiation, increased exclusively on day 28. The Runx2 expression during the differentiation of PDL cells differs in previous studies. One study reported no difference on day 7 compared to day 0, but Runx2 expression increased on day 14 [29]. In another study, Runx2 expression increased compared to the control group on days 14, 21, and 28 after differentiation [30]. Similar to this study, another study reported that Runx2 expression increased at the intermediate stages of differentiation on 3 and 7 days [31]. Similar to other studies [29, 30, 32], the pattern of increasing OCN expression as differentiation proceeded was also shown in this study. The results from this study suggest that human PDL cells obtained from commercial sources possess osteogenic potential, as indicated by the expression of osteogenic markers. These results also confirm previous finding [13] indicating the contribution of PDL cells to bone remodeling.

The expression of TRPs has been reported in human PDL cells, dental pulp cells, osteoblasts, and murine osteoblasts [5, 16, 33]. Regarding the expression of TRPCs, previous studies have investigated changes in TRPC6 expression in the osteoblast differentiation of human PDL cells and the odontogenic differentiation of human dental pulp cells [34, 35]. Wang Li et al*.* reported TRPC6 expression in the osteoblast differentiation of human PDL cells, showing high expression on days 7 and 14 and decreases on day 21 [34], similar to our results. In this study, TRPC1 was highly expressed from day 3 to day 28, TRPC3 tended to increase gradually, and TRPC4 was weakly expressed compared to other TRPCs, suggesting that various TRPCs are expressed in human PDL cells. This study is the first to evaluate the expression of various TRPCs, in addition to TRPC6, during the osteoblast differentiation of human PDL cells. Wang Li et al. showed that not only did TRPC6 expression significantly increase during osteoblast differentiation, but also TRPC6 played an important role in osteoblast differentiation of PDL cells by inhibiting and activating TRPC6 [34]. Additional evaluation through regulation of TRPCs is required to confirm that the TRPC expression identified in our findings is related to the role of TRPCs.

Next, changes in osteoclast and new bone formation during periodontitis induction were identified in mice with periodontitis. The patterns of alveolar bone loss in the ligation-induced periodontitis model indicate that alveolar bone loss increases [36], maintains [37], or recovers [38] during periodontitis induction. Mice with periodontitis in this study exhibited a pattern in which alveolar bone loss on day 7 was maintained until day 28. The number of osteoclasts increased the most on day 7 and then decreased on days 14 and 28 compared to day 7, whereas new bone increased significantly on days 14 and 28, showing the opposite pattern. This confirms that new bone formation occurs after osteoclast formation during periodontitis-induced alveolar bone loss. A previous study reported that osteoclasts continuously increased for 37 days in rats with periodontitis [39], and the different results from this study may have been due to methodological differences such as thread thickness and type. In mice with periodontitis, increased Runx2 expression in the PDL area and osteoblasts was temporarily decreased on day 14. Another study reported that Runx2 expression in PDL cells was similar to that of the control group on day 14 after periodontitis induction [40]. During periodontitis induction, the expression intensity of OCN in the PDL area increased compared to the control group, but no difference was observed in OCN expression in osteoblasts. It was also reported in a previous study that OCN expression in osteoblasts did not change during periodontitis induction [39]. New bone significantly increased from day 14 after periodontitis induction, and the expression of Runx2 and OCN increased from day 7, suggesting that osteoblast differentiation factors are increased even before new bone formation. This in vitro and in vivo study is significant because it showed that PDL cells could differentiate into osteoblasts by expressing Runx2 and OCN, and alveolar bone formation could also be affected.

TRPC channels are generally activated downstream of Gq/11-coupled receptors or receptor tyrosine kinases and are frequently proposed to act as store-operated channels (SOCs) or components of multimeric complexes that form SOCs activated by the depletion of intracellular calcium stores [41, 42]. TRPC6 is expressed in the brain, kidney, and immune and blood cells [43, 44]. A previous study evaluated the increase in TRPC6 expression in molars subjected to orthodontic force and found that appropriate mechanical force induced TRPC6 activation [45]. This study evaluated the expression of TRPC3 and TRPC6 in periodontal tissue during periodontitis induction. TRPC6 expression was increased in the PDL area and osteoblasts when periodontitis was induced, and the expression increased in osteoblasts, especially on day 28. TRPC3 expression has been reported from in vitro studies using muscle and osteoblastic cells [5, 46]. TRPC3 expression in the PDL area was increased on days 7 and 28 during periodontitis induction, and interestingly, the expression in osteoblasts was not different from that of the control group. These results suggest that TRPC6 and TRPC3 expression may be induced by periodontitis as well as by orthodontic force. Although a previous study showed that the application of mechanical force induced TRPC6 activation in the orthodontic tooth movement model [45], this study is the first to observe TRPC3 and TRPC6 expression in the periodontitis model.

This study observed an increase in the expression of TRPC3 and TRPC6 in PDL cells that were differentiating into osteoblasts and periodontitis-induced tissue but had a limitation in that the role of TRPC3 and TRPC6 in periodontitis could not be identified. Recently, TRPC has been investigated using knockout animals, and the findings suggest that TRPC channels are important in mammalian physiology and may serve as therapeutic targets. Impaired endothelial-dependent vasorelaxation in TRPC4^−/−^ mice [47, 48] and increased smooth muscle-dependent contractility in TRPC6^−/−^ mice [49] have been reported. The pancreas and salivary gland have been shown to be protected from Ca^2+^-mediated cytotoxicity in TRPC3^−/−^ mice [50]. The pharmacological inhibition of TRPC6 changed the morphology and migration speed of human PDL stem cells and mouse bone marrow mesenchymal stem cells in a specific conditional microenvironment, and TRPC6 deficiency suppressed osteoblast and osteoclast differentiation, leading to shorter orthodontic tooth movement distance [45]. In the future, changes in periodontal tissue should be observed by inducing periodontitis in TRPC3 and TRPC6 knockout animal models or by using TRPC3 and TRPC6 modulating drugs in a periodontitis animal model to clarify the functional role of TRPC3 and TRPC6 in periodontitis.

## Conclusions

This study showed that TRPC3 expression continuously increased during osteoblast differentiation and an evident increase in TRPC6 expression from the intermediate stages of differentiation. Also, TRPC6 expression increased in both the PDL area and osteoblasts in the periodontal tissue of mice with periodontitis. However, TRPC3 increased only in the PDL area. Experiments using TRPC3 and TRPC6 knockout animal models or TRPC3 and TRPC6 modulating drugs should be performed to determine how increased TRPC3 and TRPC6 expression is related to the progression of periodontitis.

## Methods

### Cell culture

Human permanent teeth-derived PDL cells were obtained from a commercial source (Catalog #2630, ScienCell, CA, USA). The PDL cells were cultured in a 100 mm dish containing growth medium at 37 °C with 5% CO_2_ until confluent on each plate. The growth medium used was Minimum Essential Medium alpha modification (Welgene, Daegu, Korea) supplemented with 10% fetal bovine serum and 1% penicillin/streptomycin. PDL cells were used at passages 4–5 in this study.

To perform the osteoblast differentiation study, two groups were designated; the control group and the differentiation group. The control group was cultured in the growth medium for 4 weeks, and the differentiation group was cultured in the osteogenic differentiation medium. The osteogenic differentiation medium was growth medium supplemented with 50 μg/mL L-ascorbic acid (Sigma-Aldrich, MO, USA) and 10 mM β-glycerophosphate (Sigma-Aldrich). The medium was replaced every 2–3 days.

### Alkaline phosphatase activity and mineralization staining

PDL cells were seeded into 24 well plates at a density of 5 × 10^4^ cells/well in the osteogenic differentiation medium. ALP activity and mineralization were estimated on days 3, 5, 7, 14, 21, and 28. To assess ALP activity, the cultured cells were washed twice with PBS and fixed in 4% paraformaldehyde solution at room temperature for 15–30 min. Then, the fixed cells were rinsed three times with deionized water and stained using the ALP staining kit (Wako, Osaka, Japan) according to the manufacturer’s protocols. The cells were reacted with 250 μL ALP staining solution for 40 min in the dark and observed by inverted microscopy (CKX41, Olympus, Tokyo, Japan). For the mineralization assay, ARS solution (Sigma-Aldrich, MO, USA) was adjusted with 0.5% NH_4_OH to pH 4.1–4.3, followed by filtration through a 0.2 μm filter. The cells were incubated in the solution for 40–60 min, dried at room temperature, washed with deionized water, and observed by inverted microscopy (CKX41, Olympus).

### Real-time PCR

The mRNA expression level of several TRPCs (TRPC1, 3, 4, and 6) and osteoblast differentiation marker genes (Runx2 and OCN) were analyzed by real-time PCR. Human PDL cells were seeded in 6 well plates at a density of 4 × 10^5^ cells/well in the growth medium. Day 0 was designated when the cells reached 80% confluence in each well. Then, the growth medium or osteogenic medium of the confluent cells was changed. To extract total RNA from the cultured cells on days 0, 1, 3, 5, 7, 14, 21, and 28, the cells were lysed with QIAzol (Qiagen, Hilden, Germany) followed by a phenol–chloroform extraction procedure. One μg of total RNA was reverse-transcribed to synthesize cDNA using a kit and oligomer dt primer (Bioneer, Daejeon, Korea). The reverse transcription reaction was performed at 42 °C for 60 min, followed by 94 °C for 5 min. Real-time PCR was performed using SYBR® Premix EX TaqTM (TaKaRa, Shiga, Japan) and StepOnePlus® Real-Time PCR System (Life Technologies, CA, USA). The PCR conditions for qPCR were as follows: 95 °C for 10 min, followed by 40 amplification cycles of 95 °C for 10 s and 54 – 60 °C for 30 s. Relative quantification was performed using the comparative 2-ΔΔCt method. The samples were normalized to glyceraldehyde 3-phosphate dehydrogenase (GAPDH) at each point. The primer sequences and annealing temperature for real-time PCR are shown in Table 1.

**Table 1:** Primers used for real-time PCR

Target	Primers (5′ to 3′)	Annealing Tm
Runx2	F: GCCTTCAAGGTGGTAGCCC R: TCCACTCCGGCCCACAAATC	60
OCN	F: TAGTGAAGAGACCCAGGCGCTA R: TCACAGTCCGGATTGAGCTCA	60
TRPC1	F: ATCAAAAGGCAAGGTCAAACGG R: ACAGATCTTGGCGCAGTTCGT	57
TRPC3	F: GCATTCTCAATCAGCCAACACG R: TCCTCAGTTGCTTGGCTCTTGT	57
TRPC4	F: ATGAGGAACCTGGTGAAGCGATA R: GCATTCGCAGATTGTATTGTGGA	54
TRPC6	F: CATTTACTGGTTTGCTCCATGCA R: GTGCTGGTTTCATTAGGAAGGAG	57
GAPDH	F: CAAATTCCATGGCACCGTCA R: GACTCCACGACGTACTCAGC	58

### Animals

Seven-week-old male C57BL/6 mice were purchased from Orient Bio (Seongnam, Korea) and housed in standard polypropylene cages in specific pathogen-free conditions on a 12-h light–dark cycle. The temperature was kept constant at 22 °C, with humidity of approximately 60%, using automatic controls. Food and water were given ad libitum. The Institutional Animal Care and Use Committee of Yonsei University approved all animal care and experimental protocols (approval numbers: 2018-0031).

### Periodontitis induction

After one week of acclimatization, the mice were divided into a control group and a periodontitis group. All mice were anesthetized with a 2:1 mixture of zoletil 50 (30 mg/kg; Virbac) and rompun (10 mg/kg; Bayer Korea). Then, periodontitis was induced by the ligature of the mandible first molars with dental floss (Oral-B, OH, USA). The periodontitis groups were euthanized using CO_2_ gas on the 7th day (D7), 14th day (D14), and 28th day (D28) after periodontitis induction. The control groups were euthanized at the same time points.

### Evaluation of alveolar bone loss and formation

The collected mandibles were fixed in 10% neutral-buffered formalin for 24 h and decalcified in 10% ethylene diamine tetra acetic acid (Deajung, Siheung, Korea) for 4 weeks. The mandibles were embedded in paraffin and sectioned sagittally at a thickness of 4 μm. Slide sections in which the dental pulp of the first molars was well-revealed were chosen. The slide sections were deparaffinized with xylene and rehydrated sequentially in an alcohol series. Slides were stained with H&E for alveolar bone and osteoid area measurements. Alveolar bone loss was measured by the alveolar bone area in the furcation and the distance of the CEJ-ABC in the distal area. The osteoid area was measured in the bright pink-colored unmineralized area between the alveolar bone surface and the osteoblasts in the region of interest (ROI). For the determination of osteoclast formation, slides were stained with TRAP stain (Wako) and methyl green (Trevigen, MD, USA) according to the manufacturer’s instructions. The number of TRAP-positive osteoclasts was analyzed by counting along the surface of the alveolar bone and then dividing it into the length of the alveolar bone in the ROI. The ROI for the alveolar bone area was evaluated in a 0.5 mm area below the top of the furcation, and the ROI for osteoclasts and osteoid formation was evaluated in a 0.4 mm area below the ABC. The slides were scanned with an Aperio AT2 Digital Whole Slide scanner (Leica Biosystems, Wetzlar, Germany) and analyzed using Aperio ImageScope software (version 12.4.0).

### Immunohistochemistry

For immunochemical staining of Runx2, OCN, TRPC3, and TRPC6, endogenous peroxidase activity was inhibited with H_2_O_2_ (3%, in methanol) for 20 min, followed by antigen retrieval with trypsin (Abcam, Cambridge, UK) for 15 min at 37 °C. To block non-specific activity, 2.5% normal horse serum (Vector Laboratories, CA, USA) was applied to the slides for 1 h at room temperature. The slides were then incubated with primary antibodies of rabbit anti-Runx2 (1:400, Biorbyt, Cambridge, UK), mouse anti-OCN (1:400, Santa Cruz, TX, USA), rabbit anti-TRPC3 (1:2000, Lsbio, WA, USA), and rabbit anti-TRPC6 (1:2000, Lsbio) overnight at 4 °C. After that, secondary antibodies of horse anti-mouse/rabbit IgG (ImmPRESS HRP Universal Antibody, Vector Laboratories) and streptavidin peroxidase (Abcam) were applied for 30 min at room temperature. Subsequently, 3,3′-diaminobenzidine chromogenic substrates were applied to the slides (Dako, CA, USA) and methyl green was used as a counterstain (R&D systems, MN, USA). The expression of Runx2, OCN, TRPC3, and TRPC6 in the PDL area was graded according to the degree of staining (0: none, 1: weak, 2: medium, and 3: strong) and the number of Runx2-, OCN-, TRPC3-, and TRPC6-positive osteoblasts was calculated by dividing the length of the alveolar bone surface in the ROI. The ROI for immunohistochemistry was evaluated in a 0.4 mm area below the ABC. The slides were scanned with an Aperio AT2 Digital Whole Slide scanner (Leica Biosystems) and analyzed using Aperio ImageScope software.

### Statistical analyses

Statistically significant differences between the two groups in the in vitro study, were analyzed by the Student’s t-test (*p* < 0.05) using PRISM 5.0 software (CA, USA). Statistical analyses for the in vivo study were performed using SPSS 22.0 software (IL, USA). A nonparametric 2-way independent test was used between the control group and the periodontitis group at each time point (*p* < 0.05). Significance levels for D7, D14, and D28 of each group were determined by a non-parametric Kruskal–Wallis test (*p* < 0.05). If the difference was significant, the Mann–Whitney *U* test (*p* < 0.017) was also applied. All data were expressed as the mean ± standard error (SE).

## Data Availability

All the data generated or analyzed during this study are included in this published article.

## Abbreviations

ABC: Alveolar bone crest
ALP: Alkaline phosphatase
ARS: Alizarin red S
CEJ: Cementoenamel junction
D7: 7th day
D14: 14th day
D28: 28th day
GAPDH: Glyceraldehyde 3-phosphate dehydrogenase
H&E: Hematoxylin and eosin
OCN: Osteocalcin
PDL: Periodontal ligament
SE: Standard error
TRAP: Tartrate-resistant acid phosphatase
TRP: Transient receptor potential
TRPA: Transient receptor potential ankyrin
TRPC: Transient receptor potential canonical
TRPM: Transient receptor potential melastatin
TRPML: Transient receptor potential mucolipin
TRPN: Transient receptor potential no mechanoreceptor potential C
TRPP: Transient receptor potential polycystin
TRPV: Transient receptor potential vanilloid
